# Productivity in Physical and Chemical Science Predicts the Future Economic Growth of Developing Countries Better than Other Popular Indices

**DOI:** 10.1371/journal.pone.0066239

**Published:** 2013-06-12

**Authors:** Klaus Jaffe, Mario Caicedo, Marcos Manzanares, Mario Gil, Alfredo Rios, Astrid Florez, Claudia Montoreano, Vicente Davila

**Affiliations:** 1 Centro de Estudios Estratégicos, Universidad Simón Bolivar, Caracas, Miranda, Venezuela; 2 Departamento de Física, Universidad Simón Bolivar, Caracas, Miranda, Venezuela; 3 Departamento de Matemáticas, Universidad Simón Bolivar, Caracas, Miranda, Venezuela; Universidad Veracruzana, Mexico

## Abstract

Scientific productivity of middle income countries correlates stronger with present and future wealth than indices reflecting its financial, social, economic or technological sophistication. We identify the contribution of the relative productivity of different scientific disciplines in predicting the future economic growth of a nation. Results show that rich and poor countries differ in the relative proportion of their scientific output in the different disciplines: countries with higher relative productivity in basic sciences such as physics and chemistry had the highest economic growth in the following five years compared to countries with a higher relative productivity in applied sciences such as medicine and pharmacy. Results suggest that the economies of middle income countries that focus their academic efforts in selected areas of applied knowledge grow slower than countries which invest in general basic sciences.

## Background

Knowledge and wealth have been recognized to be related since ancient times [1]–[4]. Napoleon used to say that “there cannot be a great nation without great mathematics”. Yet how this relationship works in the modern world is still a sensitive political issue [5]–[8]. There is no doubt that scientific and technological research affects economic development [9]–[10], for example. Scientific development and the wealth of nations are closely linked [11]. Scientific development was shown to correlate with tolerance and openness of a society, reflecting the fact that attitudes favoring science are related to valuation of empirical facts over personal convictions, which lay at the base of modern scientific progress [12]–[13]. This statistical analysis correlating scientific productivity with economic development, found that increases in economic development preceded that of scientific development, suggesting that the role of science was rather allowing sustained long term economic development but not triggering its.

A significant recent contribution to the debate was made by Hidalgo et al [14]–[16] who proposed a novel Economic Complexity Index (ECI) to account for knowledge embedded in society that produces wealth. In their words “*Modern societies can amass large amounts of productive knowledge because they distribute bits and pieces of it among its many members. But to make use of it, this knowledge has to be put back together through organizations and markets. Thus, individual specialization begets diversity at the national and global level. Our most prosperous modern societies are wiser, not because their citizens are individually brilliant, but because these societies hold a diversity of knowhow and because they are able to recombine it to create a larger variety of smarter and better products*.” This ECI reflects the composition of a country's productive output and its structures that emerge to hold and combine knowledge [16]

These results open new questions. Do certain areas of science promote economic development more than others? Are more applied sciences better in advancing economic development than more general basic sciences?

## Methods

We had no external funding sources for this study.

In order to answer these questions we first assessed the closeness of the various widely used indices for knowledge and socio-economic development to the classical index of national wealth such as Gross Domestic Product per capita (GDPc) . This was done using a Joining Tree Cluster Analysis from the sofware Statistica 7, comparing the weighted pair-group average using euclidean distance and computing a matrix from this distances . The tree was then drawn from the data in the matrix.

Then we compared the relative publication effort made by each country regarding research in different areas of knowledge, with its present and future national wealth. Data of the number of publication by area for each country for the years starting 1998 came from the database of Scopus compiled by SCImago [17], whereas data for 1982 and 1992 was compiled manually by us from the Web of Science. We calculated the relative research effort of each scientific subject area as the percentage of the total number of publications of that country published in journals of that area in a year. For example, the number of publications in mathematical journals of that country, divided by the total number of publications in all subject areas of that country, multiplied by 100, served as the estimate of relative research effort in mathematics for that country. This number was used to calculate the “Revealed Comparative Advantages” (RCA) of the scientific publication effort, adapted from the economic literature [18]. RCA is a ratio of two shares. The numerator is the share of a country's publications in a given discipline or area of science in its total number of publications. The denominator is the share of the world's number of publications in that same discipline in the total world's publications.

In order to avoid statistical pitfalls due to non-linearity in our data, we used only nonparametric statistics for the analysis of the relationship between RCA and economic growth. Only countries with more than 100 publications in 1982, or 200 in 1996, and which had their GDPc data for the required years in the World Bank database, were taken into account.

Economic wealth was estimated using the Gross National Product per capita (GDPc) as calculated by the World Bank (GDP per capita based on purchasing power parity at constant 2005 US $). Percentage growth in wealth was estimated by calculating the perceptual increase of GDPc during a given period of time.

Countries with over 100 publications in 1998 recorded by Scopus and with GDPc data provided by the World Bank were used for the present analysis. Only 101 countries fulfilled these criteria.

## Results

**Figure 1:**
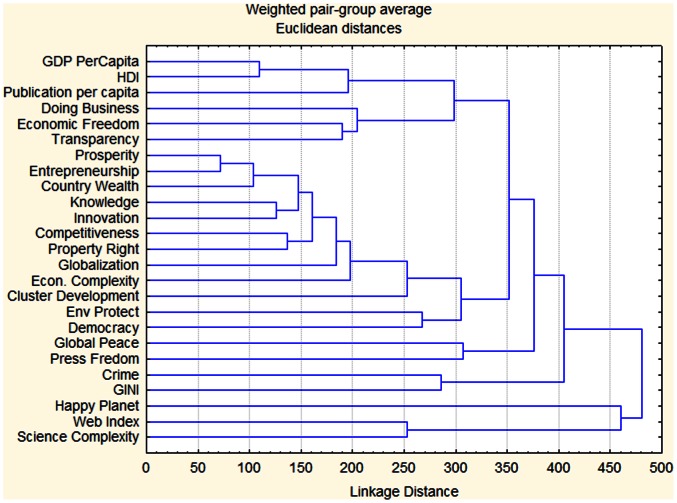
Cluster analysis of the country ranks of common econometric indices (sources for the indices are detailed in Table S1).

Scientific productivity is a much better predictor of economic wealth and Human Development of a nation than other variables tracked by a number of commonly used indices proposed worldwide. Figure 1 show that the number of publications per capita of a country (Publication) is the index closest the GDP per capita and to the Human Development Index (HDI) of the country. “Publication” correlates much stronger with the wealth per capita of a nation than any of the other indices tested.

Rich countries with high GDPc publish relatively more in certain scientific disciplines, whereas poor countries with low GDPc publish relatively more in other disciplines (Table 1). The table shows the correlations between RCA or the relative research effort in each discipline assed by the publication record of the year 2010 of each country, with its GDPc of the same year. The table shows that richer countries publish more and therefore probably invest more research effort in neurosciences, computer sciences and psychology than poorer ones; whereas poorer countries publish more research in agriculture and multidisciplinary sciences.

**Table 1:** Relation between the relative research efforts in the various disciplines in each country during 2010, and technological and economic development indicators.

Spearman correlations	ECI 2008 (n = 76)	p-level	GDPc 2010 (n = 80)	p-level
Neuroscience	0.676	0.0000	0.718	0.0000
Psychology	0.361	0.0014	0.632	0.0000
Computer Science	0.531	0.0000	0.586	0.0000
Biochemistry, Genetics & Molecular Biology	0.642	0.0000	0.569	0.0000
Arts & Humanities	0.269	0.0188	0.493	0.0000
Health Professions	0.365	0.0012	0.477	0.0000
Decision Sciences	0.231	0.0444	0.442	0.0000
Business, Management & Accounting	0.225	0.0502	0.411	0.0002
Economics, Econometrics & Finance	0.103	0.3781	0.369	0.0008
Dentistry	0.184	0.1110	0.262	0.0197
Engineering	0.308	0.0068	0.253	0.0244
Medicine	0.045	0.6993	0.204	0.0719
Social Sciences	−0.074	0.5272	0.151	0.1848
Earth & Planetary Sciences	−0.067	0.5637	0.144	0.2057
Nursing	−0.106	0.3617	0.068	0.5516
Physics & Astronomy	0.284	0.0130	0.068	0.5530
Mathematics	0.076	0.5166	0.052	0.6515
Veterinary	−0.059	0.6109	0.008	0.9470
Chemical Engineering	0.102	0.3807	0.007	0.9509
Materials Science	0.158	0.1729	−0.040	0.7249
Chemistry	0.175	0.1306	−0.059	0.6034
Energy	−0.112	0.3337	−0.133	0.2435
Immunology & Microbiology	−0.137	0.2363	−0.172	0.1306
Pharmacology, Toxicology & Pharmaceutics	−0.149	0.1975	−0.197	0.0824
Environmental Science	−0.292	0.0104	−0.240	0.0329
Multidisciplinary	−0.315	0.0056	−0.280	0.0126
Agricultural & Biological Sciences	−0.362	0.0013	−0.341	0.0021
Total Publication/capita (n = 79)	0.79	0.000	0.90	.000

The table present Spearman correlation coefficients of comparisons between RCA of publications pertaining to a given area; with technological development (ECI) and with wealth of that country (GDPc for 2010). n indicates number of countries with data for the corresponding analysis.

**Figure 2:**
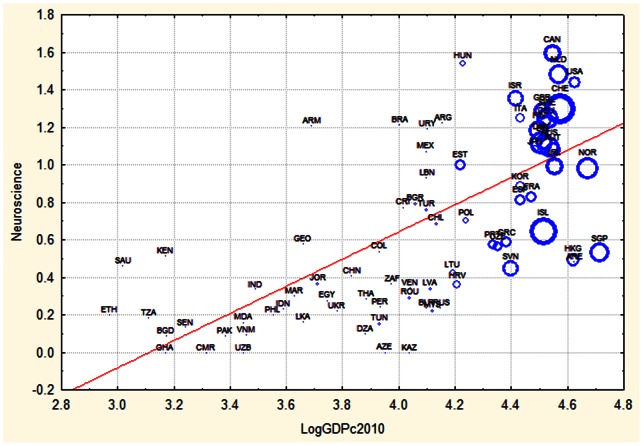
Relation between national wealth per capita (GDPc) and relative investment in neuroscience during 2010. The size of the circle is proportional to the total number of publications of that country per capita during 2010. Countries names are given with ISO abbreviations.

**Figure 3:**
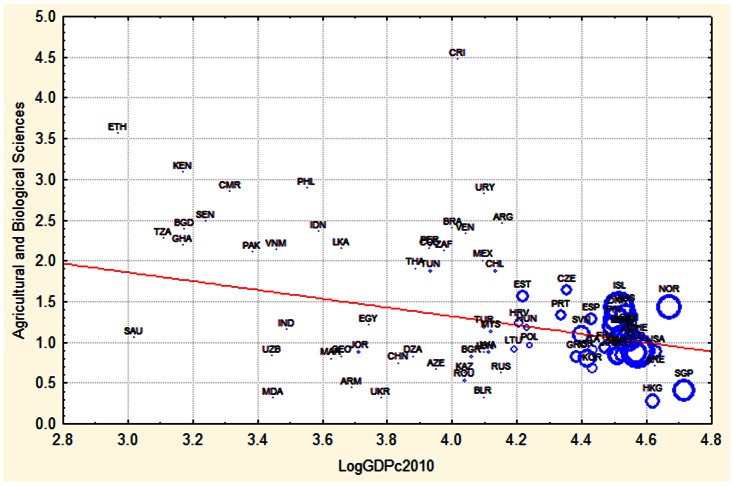
Relation between national wealth per capita (GDPc) and relative investment in agriculture during 2010. The size of the circle is proportional to the total number of publications of that country per capita during 2010. Countries names are given with ISO abbreviations.

This difference is visualized in Figure 2 and 3. We see that Canada, a high income country, is the country with the highest RCA in neurosciences, whereas Costa Rica, a medium income country, shows the highest RCA in Agriculture.

Correlations between the RCA of the publication effort of scientific disciplines during 2000 with economic growth in the following years, estimated as percent increase in GDPc during the periods 2000–2005 (Table 2) shows a different result. Here relative research efforts in physics and chemistry were the best predictors for future economic growth, and efforts in medicine and psychology the best predictors for poor future economic growth. A part, but certainly not all, of the correlation between relative productivity in physical and chemical science and future economic growth could be explained by an additional correlation with development of technological knowledge. The Economic Complexity Index, as calculated in by Hausmann et al [16], mirrors some but not all of the patterns of correlation between RCS in scientific publications and GDPc growth in the following 5 years. For example, RCA in physics and material sciences was positively correlated to both, the economic complexity index achieved 8 years later and the economic growth achieved 5 years later. RCA in chemistry, however, did not correlated significantly with economic complexity but did correlate positively with economic growth. RCA in computer science, health, biochemistry and neuroscience, for example, correlated with future economic complexity but not with economic growth.

**Table 2:** Spearman correlation coefficients of the comparisons between the relative research effort (RCA) of a given discipline in the year 2000 in a country with its economic growth as % difference in GDP per capita during the years 2000–2005.

Spearman correlations	ECI 2008-1998 (n = 61)	p-level	GDPc 2005-2000 (n = 89)	p-level
Materials Science	−0.029	0.8231	0.498	0.0000
Chemistry	−0.117	0.3713	0.439	0.0000
Physics & Astronomy	−0.132	0.3095	0.399	0.0002
Mathematics	−0.263	0.0402	0.259	0.0206
Engineering	−0.137	0.2909	0.241	0.0316
Energy	0.321	0.0117	0.176	0.1179
Chemical Engineering	−0.194	0.1346	0.139	0.2190
Earth & Planetary Sciences	0.049	0.7062	−0.024	0.8358
Decision Sciences	−0.209	0.1068	−0.050	0.6574
Business, Management & Accounting	0.046	0.7270	−0.102	0.3698
Computer Science	−0.267	0.0373	−0.122	0.2817
Health Professions	−0.277	0.0308	−0.260	0.0201
Environmental Science	0.145	0.2657	−0.272	0.0148
Dentistry	−0.106	0.4141	−0.287	0.0097
Social Sciences	0.108	0.4075	−0.301	0.0067
Agricultural & Biological Sciences	0.231	0.0729	−0.303	0.0063
Arts & Humanities	−0.102	0.4354	−0.305	0.0059
Veterinary	−0.036	0.7823	−0.328	0.0030
Economics, Econometrics & Finance	−0.094	0.4706	−0.345	0.0017
Immunology & Microbiology	0.019	0.8841	−0.382	0.0005
Biochemistry, Genetics & Molecular Biology	−0.380	0.0025	−0.403	0.0002
Pharmacology, Toxicology & Pharmaceutics	−0.148	0.2560	−0.404	0.0002
Neuroscience	−0.549	0.0000	−0.468	0.0000
Nursing	−0.147	0.2596	−0.483	0.0000
Psychology	−0.440	0.0004	−0.502	0.0000
Multidisciplinary	−0.294	0.0213	−0.504	0.0000
Medicine	−0.232	0.0719	−0.579	0.0000
Total Publication/capita (n = 80)	−0.52	0.0000	−0.31	0.006

Correlation coefficients of the comparison between RCA and economic complexity (ECI) are also presented. n indicates number of countries with data for the corresponding analysis.

**Figure 4:**
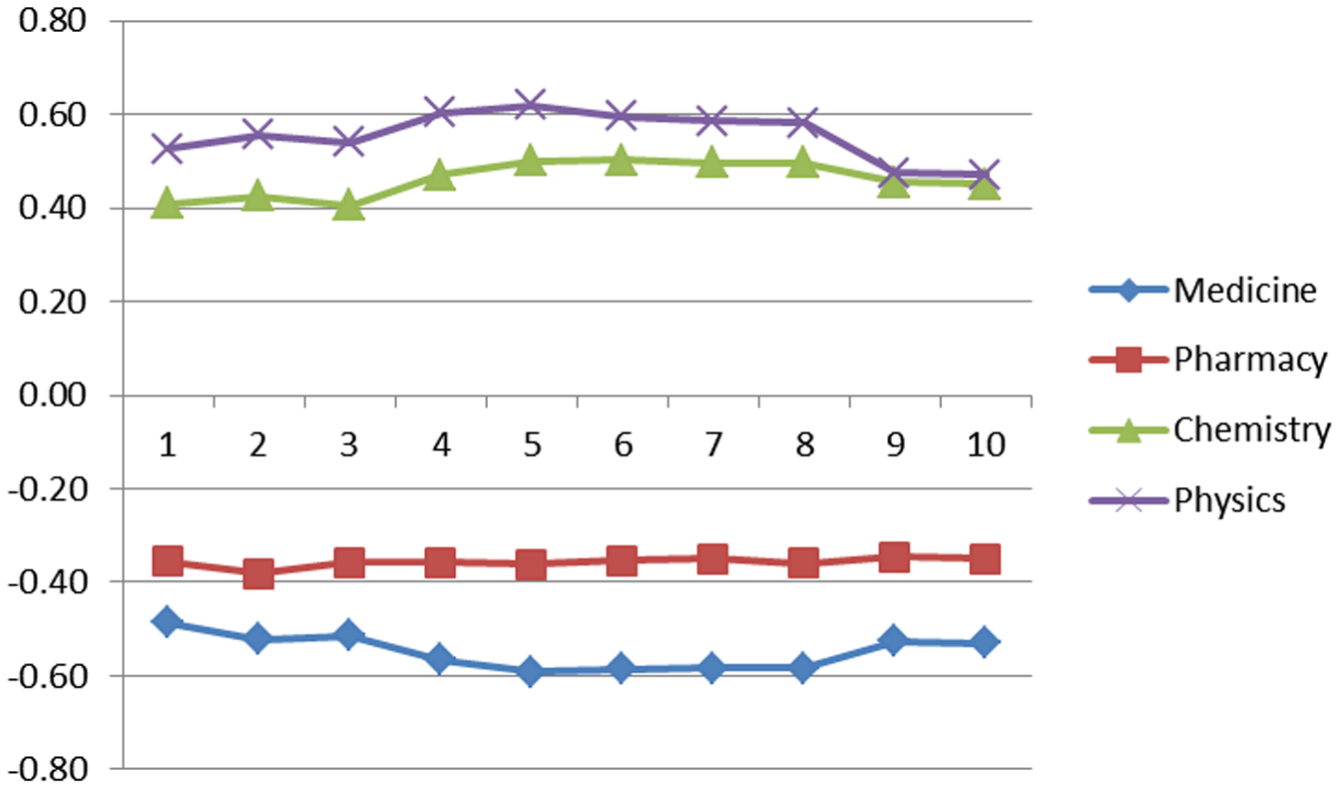
Strength of correlation between RCA of a given discipline in 2000 and the economic growth achieved by the country in the following years measured as % increase in GDPc in the years following 2000.

A finer temporal analysis showed that the highest correlation scores were obtained 5 to 7 years after the relative research effort was assessed in 2000 (Figure 4).

**Figure 5:**
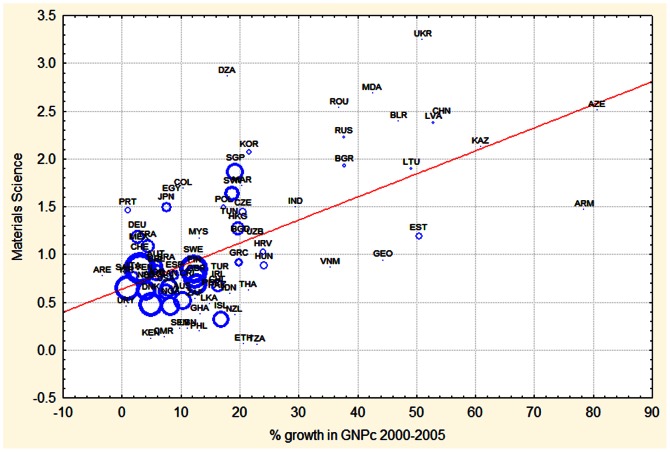
Relation between economic growth achieved during the period 2000–2005 (% change in GDPc) and RCA of physics during 2000. The size of the circles is proportional to the total number of publications per capita of that country during 2000. Countries names are given with their ISO abbreviation.

**Figure 6:**
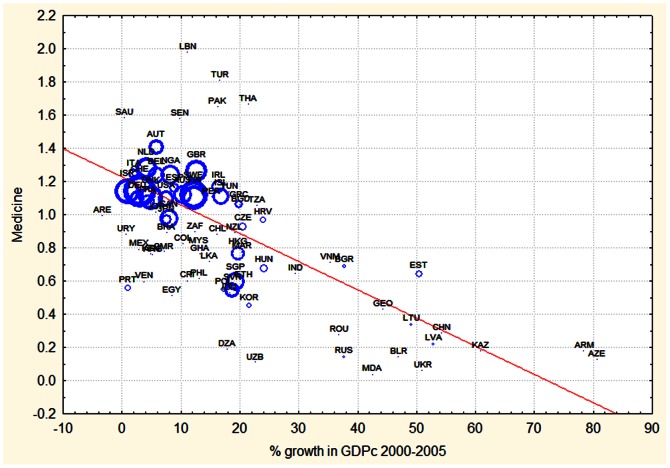
Relation between economic growth achieved during the period 2000–2005 (% change in GDPc) and RCA of medicine during 2000. The size of the circles is proportional to the total number of publications per capita of that country during 2000. Countries names are given with their ISO abbreviation.

Figures 5 and 6 illustrate the two extreme correlations revealed in Table 2. Figure 5 shows that most countries with a relative investment in material sciences (RCA>1.5) during 2000, had growth rates in the following 5 years above 30%. These countries include Armenia, Azerbaijan, Kazakhstan, Ukraine, Latvia, Belarus, Georgia, Moldova and China. The country showing the highest RCS in material sciences was Ukraine which was also among the five fastest growing countries in GDPc during 2000–2005. The outlier was Algeria with a high RCS in material sciences during the year 2000 and economic growth in the following 5 years of only about 20%. Table 3 lists the main features of each of these economies during this period as reported by the Central Intelligence Agency of the USA.

**Table 3:** Summary economic data from “The World Factbook, CIA”, for countries with the highest GDP growth between the years 2000–2010.

Azerbaijan:	Azerbaijan's high economic growth during 2006–2008 was attributable to large and growing oil exports, but some non-export sectors also featured double-digit growth, including construction, banking, and real estate, although most of this increase was tied to growth in the hydrocarbon sector.
Kazakhstan	Possesses enormous fossil fuel reserves and plentiful supplies of other minerals and metals, such as uranium, copper, and zinc. It also has a large agricultural sector featuring livestock and grain. Extractive industries have been and will continue to be the engine of this growth
Russia	Russia became the world's leading oil and gas producer
Ghana	Sound management, a competitive business environment, and sustained reductions in poverty levels. Ghana is well endowed with natural resources and agriculture
Armenia	Developed a modern industrial sector, supplying machine tools, textiles, and other manufactured goods to sister republics, in exchange for raw materials and energy
Ukraine	Fertile black soil generated more than one-fourth of Soviet agricultural output, and its farms provided substantial quantities of meat, milk, grain, and vegetables to other republics. Likewise, its diversified heavy industry supplied the unique equipment (for example, large diameter pipes) and raw materials to industrial and mining sites (vertical drilling apparatus
China	China became the world's largest exporter of industrial products
Moldova	The economy depends heavily on agriculture, featuring fruits, vegetables, wine, and tobacco. Moldova must import almost all of its energy supplies
Romania	The country emerged in 2000 from a punishing three-year recession thanks to strong demand in EU export markets
Belarus	Economic output, which had declined for several years following the collapse of the Soviet Union, revived in the mid-2000s thanks to the boom in oil prices
Bulgaria	Entered the EU on 1 January 2007, averaged more than 6% annual growth from 2004 to 2008, driven by significant amounts of bank lending, consumption, and foreign direct investment
Uganda	Has fertile soils, regular rainfall, small deposits of copper, gold, and other minerals, and recently discovered oil. Agriculture is the most important sector of the economy, employing over 80% of the work force. Coffee accounts for the bulk of export revenues

Figure 6 shows that none of the fast growing countries with a % growth in GDP per capita of more than 30% had a RCA in medicine above 0.8. Rich countries (those with large circles) had relatively high RCA in medicine, but due to the fact that advanced economies tend to grow slower than emerging ones, showed only modest growth in GDPc during the years 2000–2005. The country with the highest RCS in medicine was Lebanon which showed very poor growth in GDPc during 2000–2005.

The pattern observed for the year 2000 was not exceptional. In Table 4 we show that in different historic moments, a highly significant correlation between high RCA in science and fast economic growth in the following year can be demonstrated. RCA of physics, chemistry and material science were good predictors for future economic growth in all years except 2005. RCA of these disciplines in the year 2005 did not correlated with economic growth in the following 5 years. This lack of correlation can be explained by the global financial crisis during the last 3 years of that period which wiped out economic growth worldwide.

**Table 4:** Spearman correlation coefficients between RCA of selected disciplines in a given year and economic growth during the following 5 years using different databases and for different moments in time.

**Scopus**	**1996(85)**	**2000(80)**	**2005(82)**
Agricultural & Biological Sciences	−0.363	−0.303	0.056
Arts & Humanities	−0.063	−0.305	−0.319
Biochemistry, Genetics & Molecular Biology	0.061	−0.403	−0.370
Business, Management & Accounting	−0.115	−0.102	−0.144
Chemical Engineering	0.162	0.139	0.148
**Chemistry**	**0.342**	**0.439**	**0.233**
Computer Science	0.146	−0.122	−0.392
Decision Sciences	0.086	−0.050	−0.245
Dentistry	−0.282	−0.287	−0.312
Earth & Planetary Sciences	0.051	−0.024	−0.032
Economics, Econometrics & Finance	−0.245	−0.345	−0.359
Energy	−0.065	0.176	0.137
Engineering	0.109	0.241	−0.039
Environmental Science	−0.320	−0.272	0.005
Health Professions	0.025	−0.260	−0.433
Immunology & Microbiology	−0.235	−0.382	−0.087
**Materials Science**	**0.381**	**0.498**	**0.256**
Mathematics	0.259	0.259	−0.033
Medicine	−0.261	−0.579	−0.255
Multidisciplinary	−0.155	−0.504	0.014
Neuroscience	−0.035	−0.468	−0.557
Nursing	−0.153	−0.483	−0.408
Pharmacology	−0.092	−0.404	−0.127
**Physics**	**0.450**	**0.399**	**0.085**
Psychology	−0.071	−0.502	−0.492
Social Sciences	−0.308	−0.301	−0.140
Veterinary Sci.	−0.277	−0.328	−0.136

Correlations in colors are significant at the level p<0.01.

This pattern emerges also if a different database, such as The Web of Science, and much older data is used. For 1982 (data for 64 countries), of the 247 areas used by the Web of Science at that time to classify the journals, very few produced statistically significant (p<0.01) positive correlations between the subsequent GDPc growth in the following years and the RCA of publications in a given area. These were: Asian Studies (spearman correlation = 0.54), Physics, Fluids & Plasmas (0.51), Engineering, Manufacturing (0.42) Andrology (0.39), Social Work (0.37), Engineering, Industrial (0.34), Physics, Particles & Fields (0.34). For 1987 (data for 70 countries) not a single of these 247 areas correlated with the subsequent GDPc growth. For 1992 (data for 88 countries) only Computer Science, Theory & Methods (0.31), Economics (0.30) and Engineering, Manufacturing (0.28) correlated significantly with the subsequent 5 year GDPc growth. That is, despite the fact that the Web of Science in the decades of 1980 and 1990 compiled information about publications, based on a very reduced and selective set of journals, coming from a small group of countries, their data shows a now familiar trend: Countries with high relative investment in physics and engineering are more likely to show higher economic growth in future years.

It is tempting to postulate a direct causal relation between economic growth and the development of certain scientific areas, or vice-versa. This direct causal relationship, however, does not exist as shown in Table 5. Here we perform a temporal relation analysis inspired by Granger [19] but for data from 1996 to 2005 where the quality of the databases is comparable.

**Table 5:** Correlations between economic growth and changes in RCA of the different disciplines classified by Scopus, within simultaneous and asynchronous time intervals.

N = 85 countries	RCA 1996 to 2000 vs GDP 1996 to 2000		RCA 1996 to 2000 vs GDP 2001 to 2005		RCA 2001 to 2005 vs GDP 1996 to 2000		RCA 2001 to 2005 vs GDP 2001 to 2005	
	Spearman	p-level	Spearman	p-level	Spearman	p-level	Spearman	p-level
**Agricultural and Biological Sciences**	0.07	0.552	−0.10	0.344	0.15	0.165	0.09	0.430
**Arts and Humanities**	0.10	0.346	0.00	0.992	−0.11	0.319	−0.05	0.618
**Biochemistry, Genetics and Molecular Biology**	0.06	0.589	−0.02	0.883	−0.16	0.139	0.20	0.069
**Business, Management and Accounting**	0.06	0.615	0.17	0.123	0.16	0.153	−0.02	0.862
**Chemical Engineering**	−0.14	0.215	0.11	0.319	−0.16	0.144	−0.01	0.912
**Chemistry**	0.01	0.899	−0.13	0.221	−0.15	0.160	0.00	0.974
**Computer Science**	−0.17	0.125	**−0.34**	**0.001**	0.01	0.957	−0.11	0.296
**Decision Sciences**	−0.08	0.493	−0.07	0.498	−0.16	0.133	−0.16	0.153
**Dentistry**	0.02	0.844	0.16	0.139	0.00	0.987	0.10	0.378
**Earth and Planetary Sciences**	**0.24**	**0.027**	−0.08	0.469	−0.06	0.569	−0.20	0.066
**Economics, Econometrics and Finance**	0.11	0.301	−0.02	0.876	−0.03	0.789	−0.06	0.558
**Energy**	0.00	0.997	−0.20	0.069	0.18	0.108	0.06	0.575
**Engineering**	0.06	0.604	0.02	0.834	−0.02	0.821	−0.11	0.301
**Environmental Science**	0.09	0.436	**−0.23**	**0.038**	0.15	0.182	0.11	0.331
**Health Professions**	−0.07	0.514	0.07	0.536	−0.06	0.593	−0.14	0.210
**Immunology and Microbiology**	0.12	0.270	0.21	0.056	−0.20	0.065	−0.11	0.306
**Materials Science**	−0.11	0.334	**−0.23**	**0.036**	**−0.22**	**0.043**	−0.03	0.757
**Mathematics**	−0.20	0.064	0.06	0.608	0.06	0.608	**−0.28**	**0.011**
**Medicine**	0.01	0.942	0.12	0.268	0.03	0.757	0.16	0.145
**Neuroscience**	0.17	0.115	0.11	0.310	**−0.25**	**0.021**	−0.17	0.123
**Pharmacology, Toxicology and Pharmaceutics**	−0.05	0.670	0.03	0.818	−0.10	0.351	**0.28**	**0.008**
**Physics and Astronomy**	−0.18	0.102	−0.08	0.453	−0.16	0.131	−0.03	0.751
**Psychology**	−0.02	0.827	−0.01	0.907	0.16	0.134	−0.17	0.117
**Social Sciences**	0.08	0.472	−0.12	0.258	0.09	0.416	−0.08	0.488
**Veterinary**	0.04	0.749	0.12	0.277	0.13	0.235	0.08	0.467
**Total number of papers in Scopus**	−0.06	0,59	0,01	0,96	−0.10	0.38	0.17	0,11

Table 5 compares changes in RCA and GDP during the same period of time and between 5 year periods just before or after the other. Thus, if GDP growth would foment RCA in basic science with a time lag, or vice versa, significant correlations should appear in the asynchronous comparison of time intervals. If both RCA growth and GDP growth are triggered by the same variable, both should correlate if compared in the same time period. Results show no significant correlations at all. That is, as the data in the table represents a Multiple Comparisons Problem, we used the Bonferroni Correction, complemented with the technique for False Discovery Rate [20], which calculates the likely fraction of false positives in relation to the total number of statistical comparisons. Our null hypothesis is that the variables compared with the Spearman correlation test are independent. The likelihood of rejecting this hypothesis wrongly using the False Discovery Rate is 2% (p<0.02) for the correlations with the lowest p values in Table 5, in contrast to the data in the Tables 1,2 and 4; where the corrected p levels were order of magnitude lower.

## Discussion

The present analysis allows drawing the following conclusions.

1. For historical periods with no global financial catastrophes, the economic growth of middle income countries can be predicted with high accuracy by looking at their relative academic productivity in physical sciences and engineering.
2. Academic productivity is a much better predictor of future economic growth than economic complexity as measured in [16]. Scientific productivity is more accurate in predicting economic growth and wealth, than economic complexity. If we accept that “science is the mother of technology”, i.e. supports technological development, then science affects other aspects of live such as services, governability, rational thinking, attitudes, etc. and of the economy besides technological development [12], [23]. This result is congruent with other statistical analyses comparing the information content of statistical models using ECI with those using scientific productivity to predict economic growth [24].
3. No country with exclusive preferential investment in technology, without investment in basic science, achieved relatively high economic development. Thus, technology without science is unlikely to be sustainable.
4. The effect on the economy of scientific development is long term. It can be observed in 5 years' time. This time period is very short in terms of the process by which science creates new technology. Thus, we might be measuring the effect of science in preparing new technology leaders and in instilling rational thinking in the leaders of a country rather than the production of novel technology in middle income countries.
5. No direct correlation between development in basic science and economic growth, or vice versa, exists. We suggest that the effect mentioned in point 1 is possible the outcome of the fact that relative investment in basic science is a reliable indicator of a rational decision making atmosphere, and if other factors allow, promotes economic growth.

We have to remark that the present study excluded countries with low scientific productivity, which include all poor countries. Previous studies [12] showed that the correlation between science and wealth of a country appears only after a threshold of economic development has been reached and that a rapid increase in scientific productivity was normally observed after a previous increase in economic development. On the other hand, the relative effort to support academic activity in rich countries seems to be close to the maximum tolerated by society. Rich countries have completed their scientific and industrial revolution in the past and focus now on other aspects of the wellbeing of their citizens, as they have to manage low economic growth. This would explain the low correlations found between scientific publications and future economic growth in rich countries. Therefore, the present conclusions are valid only for middle income countries.

Jeffry Sachs [11] recommended health, energy, agriculture, climate and ecology as the areas of science where investments were most likely to promote economic growth. None of them came out as positively correlated here. On the contrary, countries that knowingly or unknowingly complied with Sachs's recommendations achieved very poor economic growth. It is investment in hard sciences and basic sciences, such as physics and chemistry that correlate strongest with economic growth. Material sciences are normally considered to be part of physics although Scopus computes the publication in this area separately.

Our results show that the correlations between basic natural science and economic development is not due to direct causal chains. This is in agreement with more recent empirical explorations in economics [21] that revealed an intricate network of reciprocal relationships between knowledge, services, environment and finance. Here we propose that scientific development works in an analogous way, affecting multiple aspects of the economy and in turn being affected by many of these aspects producing positive feedback cycles. Hirschman [22] postulated the high development theory, as the view that development is a virtuous circle driven by external economies – that is, that modernization breeds modernization. Some countries, according to this view, remain underdeveloped because they have failed to get this virtuous circle going, and thus remain stuck in a low level trap. Our data would support the proposition that investing in basic scientific research seem to be the best way a middle income country can foment fast economic growth, triggering Hirschman's virtuous cycle. This proposition is also used by Lin [25] to solve the Needham Puzzle: Why the Industrial Revolution did not originate in China. The scientific revolution needs a profound conceptual revolution which is achieved by the development of basic natural sciences [13].

As for the future, the ranking of RCA in 2010 showed that the countries with an RCA value in Physics above 2.0 were Armenia, Ukraine, Moldova, Uzbekistan, Russia, Belarus, Bulgaria, Kazakhstan and Georgia. Most of them are among the fastest growing economies in 2012. Regrettably, no country from Africa or Latin America is on this list, although Mexico and Puerto Rico, the champions in RCA values in Physics in Latin American, are the only ones with values above 1.0.

## Supporting Information

Table S1 Source of economic indicators used for the cluster analysis in Table 1. Data were from the most recent years available, spanning from 2012 to 1010.
(DOC)
