# Supplementary material for: Productivity in Physical and Chemical Science Predicts the Future Economic Growth of Developing Countries Better than Other Popular Indices
Source: PLoS One. 2013 Jun 12;8(6):e66239. doi: 10.1371/journal.pone.0066239 (PMC3680384; doi:10.1371/journal.pone.0066239)
Supplement: Table S1 — Source of economic indicators used for the cluster analysis in Table 1. Data were from the most recent years available, spanning from 2012 to 1010. (DOC) [file pone.0066239.s001.doc]

| **Abbreviation** | **INDICATOR** | **PUBLISHED BY** | **URL** |  |  |  |  |  |  |
| --- | --- | --- | --- | --- | --- | --- | --- | --- | --- |
| Cluster Develop | Cluster Development Index | INSEAD y WIPO | www.globalinnovationindex.org/gii/main/fullreport/files/Chap4/5/5.2.2.pdf | | | | | | |
| Transparency | Corruption Perception Index CPI | Transparency International | www.transparency.org/research/cpi/overview | | | | | | |
| Country Risk | Country Risk | Euromoney | www.euromoneycountryrisk.com/ | | | | | | |
| Country Wealth | Country Wealth Index | Unicef & Mics | www.measuredhs.com/ | | | | | | |
| Crime | Crime Rate Indicator | The World Bank | data.worldbank.org/indicator | | | | | | |
| Democracy | Democracy Index | The Economist Intelligence Unit | graphics.eiu.com/PDF/Democracy_Index_2010_web.pdf | | | | | | |
| Doing Business | Doing Business | World Bank y IFC | www.doingbusiness.org | | | | | | |
| Econ Complex | Economic Complexity Index ECI | Harvard Kennedy School & MIT | atlas.media.mit.edu/media/atlas/pdf/HarvardMIT_AtlasOfEconomicComplexity_Part_I.pdf | | | | | | |
| Econ Freedom | Economic Freedom | The Heritage Foundation | www.heritage.org/index/ | | | | | | |
| Env Protect | Environment Protection Index EPI | Yale Center for Environmental Law and Policy | epi.yale.edu/epi2012/rankings | | | | | | |
| Freedom Press | Press Freedom Index | Reporters without Frontiers | www.rsf-es.org/ | | | | | | |
| GDP PerCapita | GDP per Capita Ranking | World Bank. See methods | data.worldbank.org/indicator | | | | | | |
| GINI | GINI Indicator | UN Statistics | www.nationmaster.com/red/graph/eco_inc_equ_un_gin_ind‐income‐equality‐un‐gini‐inde | | | | | | |
| Global Competi | Global Competitive Report | World Economic Forum | www.weforum.org/issues/global-competitiveness | | | | | | |
| Entrepreneur | Global Entrepreneurship Monitor GEM | Babson College & London School of Economics | www.gemconsortium.org/ | | | | | | |
| Global Peace | Global Peace Index GPI | Institute for Economics and Peace 2012 | economicsandpeace.org/ | | | | | | |
| Globalization | Globalization Index GI | Foreign Policy y A.T. Kearney. | www.ForeignPolicy.com | | | | | | |
| Happy Planet | Happy Planet Index | New economic Foundation NEF | www.happyplanetindex.org | | | | | | |
| Innovation | Global Innovation Index GI | INSEAD y WIPO | www.globalinnovationindex.org/gii/ | | | | | | |
| Knowledge | Knowledge Index | The World Bank | info.worldbank.org/etools/kam2/KAM_page5.asp | | | | | | |
| Property Right | Property right Index | The International Index of Property Rights IPRI | www.internationalpropertyrightsindex.org/ | | | | | | |
| Prosperity | Prosperity Index PI | Legatum Institute | www.prosperity.com/ | | | | | | |
| Publication | Scientific Publications per capita | Scopus. See methods | This paper | | | | | | |
| Sci Complexity | Science Complex Index | See methods | This paper | | | | | | |
| Web Index | Web Index | World Wide Web Foundation | www.webfoundation.org/ | | | | | | |
| HDI | Human Development Index HDI | UNDP- United Nations Development Programme | hdr.undp.org/en/statistics/ | | | | | | |
